# Ebola Virus Infection among Western Healthcare Workers Unable to Recall the Transmission Route

**DOI:** 10.1155/2016/8054709

**Published:** 2016-11-27

**Authors:** Stefano Petti, Carmela Protano, Giuseppe Alessio Messano, Crispian Scully

**Affiliations:** ^1^Department of Public Health and Infectious Diseases, Sapienza University, Rome, Italy; ^2^University College London, London, UK

## Abstract

*Introduction.* During the 2014–2016 West-African Ebola virus disease (EVD) outbreak, some HCWs from Western countries became infected despite proper equipment and training on EVD infection prevention and control (IPC) standards. Despite their high awareness toward EVD, some of them could not recall the transmission routes. We explored these incidents by recalling the stories of infected Western HCWs who had no known directly exposures to blood/bodily fluids from EVD patients.* Methodology.* We carried out conventional and unconventional literature searches through the web using the keyword “Ebola” looking for interviews and reports released by the infected HCWs and/or the healthcare organizations.* Results.* We identified fourteen HCWs, some infected outside West Africa and some even classified at low EVD risk. None of them recalled accidents, unintentional exposures, or any IPC violation. Infection transmission was thus inexplicable through the acknowledged transmission routes.* Conclusions.* We formulated two hypotheses: inapparent exposures to blood/bodily fluids or transmission due to asymptomatic/mildly symptomatic carriers. This study is in no way intended to be critical with the healthcare organizations which, thanks to their interventions, put an end to a large EVD outbreak that threatened the regional and world populations.

## 1. Introduction

The West-African Ebola virus disease (EVD) 2014 outbreak was longer and more impactful than previous Central-African outbreaks, covering the largest geographic area, spreading to eight countries across three continents and lasting over 22 months [1]. Healthcare workers (HCWs) bore the brunt of the outbreak with high morbidity and mortality, were more likely to be infected than people in the general population, and accounted for 5% of all EVD cases [2, 3]. Nosocomial transmission was principally due to the incorrect triage of EVD patients and limited or no training on and limited availability and inappropriate use of personal protective equipment (PPE). HCWs thus did not always provide appropriate care to EVD patients, and some were infected [2]. In general, infections occurred because of low compliance with infection prevention and control (IPC) procedures.

The massive intervention of WHO and governmental and nongovernmental healthcare organizations from all over the world, with resources and “train-the-trainer” policies, improved this situation and the infectious risk among all HCWs decreased considerably, thus demonstrating that EVD infections among HCWs were preventable [2, 4, 5].

Nevertheless, some HCWs still got infected and, among them, some HCWs were from Western countries. These subjects likely complied with IPC standards, were aware of the burden of the EVD epidemics, and had also less social contacts with the members of the communities where they were active [2].

Even before the occurrence of the 2014 West-African outbreak, episodes of EVD transmission to HCWs from Western countries had been reported.

In November 1976 the first case of a HCW who developed EVD outside Africa occurred, in a laboratory worker who accidentally pricked his thumb while transferring homogenized liver from a guinea-pig infected with Ebola virus. Following the standard safety protocol, he immediately removed his gloves and immersed his damaged thumb in hypochlorite solution, then squeezing it vigorously. There was no bleeding and careful examination did not reveal a puncture wound. Nevertheless, he became EVD infected and was treated with convalescent serum and within two weeks he showed symptom remission and Ebola virus was no longer detected in blood, urine, or faeces. Therefore, he was discharged from isolation, but Ebola virus persisted in his semen for months [6].

In November 1989, during an Ebola-Reston virus outbreak among monkeys in the Philippines, some infected animals were imported into the US, where five animal handlers were daily exposed to these animals and four workers became infected without symptoms. One of them recalled cutting his finger while performing a necropsy on an infected animal, but among the remaining infected staff no transmission route was identified [7].

In 1995, during an Ebola virus outbreak in Kikwit (Democratic Republic of the Congo), four Italian missionary nurses developed EVD and three died (http://www.nytimes.com/1995/05/12/world/virus-kills-a-3d-italian-nun-at-zaire-hospital.html). There were no specific EVD-transmission-based precautions in place at that time and transmission through percutaneous exposures to infectious blood was supposed but not confirmed [8].

These episodes suggest that the events that led to Ebola virus infection could not always be known or recalled. Thus, the aim of this study was to report the anecdotal stories of some incidents occurring during the West-African EVD outbreak to HCWs from Western countries who did not recall any obvious contact of broken skin or mucous membranes with blood/bodily fluids of infected people or with surfaces and materials contaminated with these fluids, which are the acknowledged routes of human-to-human Ebola virus transmission (http://www.who.int/mediacentre/factsheets/fs103/en) (http://www.cdc.gov/vhf/ebola/healthcare-us/hospitals/infection-control.html). This study is not a field investigation or an epidemiologic study but was based on the interviews released by the EVD victims and/or the reports provided by the healthcare organizations. This was not a systematic review, and there could be other episodes of infected Western HCWs who could not recall the events that led to transmission; however, these stories could help formulate hypotheses regarding the existence and, possibly, the nature of alternative transmission routes.

## 2. Methodology

In order to collect as much material as possible, we used conventional and unconventional literature searches using the keyword “Ebola” looking for interviews and reports released by infected HCWs and/or the healthcare organizations from Western countries during the West-African outbreak.

An orthodox literature search used the databanks PubMed and Scopus. Full texts of relevant articles, according to titles, abstracts, and keywords, were collected.

An unconventional article search was performed through the Internet search engines (Google and Yahoo) using the search terms “Ebola” and “healthcare worker”/“healthcare provider”/“health worker” or the actual names of the known infected HCWs. Much of the retrieved material had been constructed from interviews with the infected HCWs obtained from the healthcare organizations and from magazines and newspapers.

The list of infected HCWs from Western countries amassed was then reduced to cases of unknown/unrecalled transmission routes in order to shed light over stories that may help provide a direction to the future research regarding Ebola virus transmission, rather than to evaluate EVD risk factors, case-fatality, incidence, or mortality rates.

## 3. Results

The searches provided the stories of fourteen Western HCWs (Table 1). Although the reported material is public, their names along with the names of the healthcare organizations are not displayed.

- Three HCWs (A, B, and C) from the USA were infected in Liberia between July and August 2014. HCW-A, a doctor caring for EVD patients, fell ill on 23 July 2014 and EVD was diagnosed four days later. No information regarding potential routes of transmission is available. HCW-B, a nurse who had no direct contact with EVD patients but assisted HCWs with donning and doffing PPE and performing decontamination, was diagnosed with EVD on 27 July (http://www.nbcnews.com/storyline/ebola-virus-outbreak/another-american-doctor-infected-ebola-charity-says-n193911) [9]. HCW-C, an obstetrician working in Liberia, did not care for EVD patients but was delivering babies following EVD-targeted prevention protocols (http://www.dailymail.co.uk/news/article-2745037/Third-U-S-missionary-51-infected-deadly-Ebola-virus-West-Africa-arrives-Nebraska-treatment.html).
- HCW-D, a UK nurse infected in Sierra Leone on 24 August 2014, spent five weeks caring for EVD patients. He and his colleagues could not recall any specific accident that might lead to Ebola virus transmission (http://www.telegraph.co.uk/news/worldnews/ebola/11072041/Ebola-victim-William-Pooley-discharged-from-hospital.html).
- HCW-E and HCW-F from Spain became infected Between August and September 2014 in Sierra Leone and were repatriated to Spain, where they died from EVD on 12 August (HCW-E) and 26 September (HCW-F). No information is provided regarding transmission (http://www.theguardian.com/world/2014/aug/12/ebola-spanish-missionary-dies-madrid-liberia) (http://www.thelocal.es/20140926/madrid-spanish-missionary-dies-from-ebola-spain).
- HCW-G was a nurse infected at a Spanish Hospital complex on 6 October 2014 while caring for one of the above two HCWs (HCW-E and HCW-F). She was the first HCW known to be infected outside of West Africa. Paradoxically, during the field investigation, among the 117 HCWs who had participated in the care of the two infected HCWs, HVW-G was classified as a “low-risk contact.” In addition, she had always used appropriate PPE such as waterproof long-sleeved clothing covering the feet, waterproof footwear, hood, face mask or goggles, double layer of gloves, and FP3 respirator and did not recall any accident during PPE use. Questioned as to how she may have contracted EVD, HCW-G replied “I really can't say, I haven't the slightest idea”(http://www.independent.co.uk/news/world/europe/spanish-nurse-teresa-romero-ramos-followed-all-protocols-and-has-no-idea-how-she-contracted-ebola-9781373.html) [10].
- HCW-H was one of hundreds of Cuban doctors sent as to Sierra Leone. WHO provided rigorous safety training and mentoring to the Cuban Medical Brigade and other foreign medical teams before starting work in the Ebola treatment centres. While working there, HCW-H contracted EVD but declared “I don't know how I got infected. There was no violation of protocols” (http://www.who.int/features/2015/cuban-doctor-survivor/en/).
- HCW-I and HCW-J were US nurses who cared for an EVD patient arriving from Liberia on 28 September 2014 who died from EVD on 8 October in Dallas. HCW-I and HCW-J became infected on 11 and 14 October, respectively, but did not report unprotected exposures with the index patients and had been categorized as low EVD risk. How these two HCWs contracted Ebola virus infection remains unknown [11]. They complained, however, of having being obliged to have worked at the hospital in chaotic surroundings with ill-prepared nurses who received little guidance on how to treat Ebola and protect themselves (http://res.dallasnews.com/interactives/nina-pham/).
- HCW-K was a US doctor, who treated EVD patients in Guinea. On 23 October 2014, nine days after returning to the US, he was diagnosed with EVD. He reported using all the prescribed PPE without any known breach [12]. His charity investigation on the transmission route was inconclusive. HCW-K conjectured that the Ebola virus could be trapped in a wet respiratory mask or that transmission occurred the day he was accidentally poked in his eye by the gloved finger of a hygienist without visible traces of human biological fluids or when he was feeding and cleaning a severely ill patient with EVD (http://nymag.com/daily/intelligencer/2015/06/craig-spencer-after-ebola.html#).
- HCW-L, a doctor from Italy working in Sierra Leone, had an episode of vomiting and diarrhoea with low fever on 20 November 2014, testing positive for Ebola virus four days later. He was repatriated to Rome, hospitalized, and treated [13]. Both the employer charity investigation and HCW-L reported that he had been observing correct procedures when treating infected patients (http://www.bbc.com/news/world-africa-30180345) (details in Italian at http://www.ilpost.it/2015/02/10/fabrizio-pulvirenti/).
- HCW-M was a UK nurse who cared for EVD patients in Sierra Leone. On December 2014, on return home to Scotland, she became ill and was diagnosed with EVD (http://www.theguardian.com/world/2014/dec/30/uk-ebola-patient-named-pauline-cafferkey- scottish-nurse). She was transferred to London for care. A joint investigation performed by the charity and the Centre for Infectious Disease Surveillance and Control, Public Health England concluded that there was no conclusive evidence about when or how she contracted EVD including the possibility of infected social contacts. According to her employing charity, “We will never be 100% sure how HCW-M contracted Ebola” (http://www.savethechildren.org.uk/sites/default/files/images/Significant_Event_Review_Summary_Findings.pdf).
- HCW-N was an Italian nurse working in Sierra Leone assisting EVD patients. Two days after his return to Italy he fell ill and, two days later, on 12 May 2015, was diagnosed with Ebola virus infection. In an interview he reported to have no idea on how transmission may have occurred (http://www.sardiniapost.it/cronaca/stefano-marongiu-infermiere-guarito-da-ebola-non-mi-sono-mai-sentito-solo/ in Italian)

Of the fourteen reported Ebola virus-infected HCWs from Western countries only two died from EVD. The reason is almost certain: all the infected HCWs were repatriated to USA or Europe, hospitalized, and underwent intensive care treatments [9, 10, 13]. Indeed, according to HCW-K, “whereas in Guinea I took care of 30 patients, in the US 30 doctors took care of me” (http://nymag.com/daily/intelligencer/2015/06/craig-spencer-after-ebola.html#).

## 4. Discussion

The common characteristics of the reported stories of these HCWs from Western countries are that, excluding the single case of HCW-D, whose colleagues suspected that he made mistakes with transmission precautions due to overworking, none of the remaining HCWs recalled any event that might lead to Ebola virus transmission (Table 1) and epidemiologic surveys failed to detect the transmission routes. Astonishingly, some of these Western HCWs, such as HCW-B and C, were not involved in the care of EVD patients. In addition, HCWs were aware, trained, and equipped to prevent Ebola virus infection transmission. We already demonstrated that relatively high disease knowledge and awareness do not necessarily lead to effective disease control and highlighted that this apparent contrast is for reasons beyond the efficacy of control measures but involve social, cultural, and behavioural dimensions [14–17].

The observation that trained and equipped HCWs could not recall the event that led to Ebola virus infection transmission leaves room for two major hypotheses.

Ebola virus transmission could be due to inapparent exposures to infected blood or bodily fluids, an acknowledged transmission route for other blood-borne pathogens, such as HCV and HBV [18, 19].

Ebola virus transmission could be due to caring for asymptomatic or mildly symptomatic EVD carriers, an hypothesis corroborated by several elements. First, Ebola virus may persist at immunologically protected sites in the body including semen, vaginal fluids, sweat, aqueous humor, urine, and breast milk of convalescent patients for months and, indeed, some convalescent patients have transmitted the infection, suggesting an important role of convalescent carriers in EVD persistence within the community [20]. Second, in endemic areas as many as 1–6% apparently healthy individuals show high levels of serum anti-Ebola virus antibodies, while 3–9% close family contacts of EVD patients are infected without developing typical EVD symptoms [21]. Third, mildly symptomatic subjects could also have played a role in infection transmission. Indeed, their prevalence during the West-African outbreak largely increased and most EVD survivors developed nonspecific EVD symptoms rather than haemorrhagic disease [22].

As already reported, this is not an epidemiologic investigation nor an inspection, and therefore, it is not possible to confirm that the EVD transmission route in these individuals was truly through inapparent exposure and/or due to asymptomatic/mildly symptomatic carriers. Even the more accurate investigations during the EVD outbreak failed to identify the exposures in more than one-fourth of infected patients [4] and, more broadly, in all communicable disease outbreaks there is always an important fraction of infected subjects with unknown or unrecalled exposures [23].

Perhaps, knowledge about human-to-human EVD transmission will improve thanks to experimental studies and to observational studies focusing on infected individuals without any obvious exposure [24], but the challenge is exactly this, distinguishing between unrecalled and unknown exposures to EVD.

## Figures and Tables

**Table 1:** Profiles of HCWs from Western countries infected with Ebola virus during the West-African outbreak who could not recall the route of EVD transmission.

HCW (country)	HCW category	Country of acquisition/diagnosis	Country of hospitalization	Probable transmission route
A (US)	Doctor	Liberia	US	N/A
B (US)	Nurse	Liberia	US	Did not care for EVD patients
C (US)	Obstetrician	Liberia	US	Did not care for EVD patients
D (UK)	Nurse	Sierra Leone	UK	Used the prescribed PPE. Mistakes in precautions due to overwork (colleagues' opinion)
E (Spain)^a^	N/A	Sierra Leone	Spain	N/A
F (Spain)^a^	N/A	Sierra Leone	Spain	N/A
G (Spain)	Nurse	Spain	Spain	Classified as low Ebola risk. Used the prescribed PPE
H (Cuba)	Doctor	Sierra Leone	Switzerland	Used the prescribed PPE
I (US)	Nurse	US	US	Classified as low Ebola risk (complained ill-prepared hospital staff)
J (US)	Nurse	US	US	Classified as low Ebola risk (complained ill-prepared hospital staff)
K (US)	Doctor	Guinea (acquisition), US (diagnosis)	US	Used the prescribed PPE
L (Italy)	Doctor	Sierra Leone	Italy	Used the prescribed PPE
M (UK)	Nurse	Sierra Leone (acquisition), UK (diagnosis)	UK	Used the prescribed PPE
N (Italy)	Nurse	Sierra Leone (acquisition), Italy (diagnosis)	Italy	Used the prescribed PPE

^a^Died from EVD.
